# Elevated Free Phosphatidylcholine Levels in Cerebrospinal Fluid Distinguish Bacterial from Viral CNS Infections

**DOI:** 10.3390/cells10051115

**Published:** 2021-05-06

**Authors:** Amani Al-Mekhlafi, Kurt-Wolfram Sühs, Sven Schuchardt, Maike Kuhn, Kirsten Müller-Vahl, Corinna Trebst, Thomas Skripuletz, Frank Klawonn, Martin Stangel, Frank Pessler

**Affiliations:** 1 Biostatistics, Helmholtz Centre for Infection Research, 38124 Braunschweig, Germany; Amani.Al-Mekhlafi@helmholtz-hzi.de (A.A.-M.); frank.klawonn@helmholtz-hzi.de (F.K.); 2 PhD Programme Epidemiology, Hannover Medical School, 30625 Hannover, Germany; 3 Department of Neurology, Clinical Neuroimmunology and Neurochemistry, Hannover Medical School, 30625 Hannover, Germany; suehs.kurt-wolfram@mh-hannover.de (K.-W.S.); Trebst.Corinna@mh-hannover.de (C.T.); skripuletz.thomas@mh-hannover.de (T.S.); stangel.martin@mh-hannover.de (M.S.); 4 Fraunhofer Institute for Toxicology and Experimental Medicine (ITEM), 30625 Hannover, Germany; sven.schuchardt@item.fraunhofer.de; 5 Research Group Biomarkers for Infectious Diseases, TWINCORE Centre for Experimental and Clinical Infection Research, 30625 Hannover and Helmholtz Centre for Infection Research, 38124 Braunschweig, Germany; maike.kuhn@t-online.de; 6 Research Core Unit Metabolomics, Hannover Medical School, 30625 Hannover, Germany; 7 Department of Psychiatry, Social Psychiatry and Psychotherapy, Hannover Medical School, 30625 Hannover, Germany; Mueller-Vahl.Kirsten@mh-hannover.de; 8 Cluster of Excellence RESIST (EXC 2155), Hannover Medical School, 30625 Hannover, Germany; 9 Centre for Individualised Infection Medicine, 30625 Hannover, Germany

**Keywords:** biomarker, central nervous system, cell membrane, enterovirus, herpes simplex virus, infection, meningitis, metabolism, varicella zoster virus

## Abstract

The identification of CSF biomarkers for bacterial meningitis can potentially improve diagnosis and understanding of pathogenesis, and the differentiation from viral CNS infections is of particular clinical importance. Considering that substantial changes in CSF metabolites in CNS infections have recently been demonstrated, we compared concentrations of 188 metabolites in CSF samples from patients with bacterial meningitis (*n* = 32), viral meningitis/encephalitis (*n* = 34), and noninflamed controls (*n* = 66). Metabolite reprogramming in bacterial meningitis was greatest among phosphatidylcholines, and concentrations of all 54 phosphatidylcholines were significantly (*p* = 1.2 × 10^−25^–1.5 × 10^−4^) higher than in controls. Indeed, all biomarkers for bacterial meningitis vs. viral meningitis/encephalitis with an AUC ≥ 0.86 (ROC curve analysis) were phosphatidylcholines. Four of the five most accurate (AUC ≥ 0.9) phosphatidylcholine biomarkers had higher sensitivity and negative predictive values than CSF lactate or cell count. Concentrations of the 10 most accurate phosphatidylcholine biomarkers were lower in meningitis due to opportunistic pathogens than in meningitis due to typical meningitis pathogens, and they correlated most strongly with parameters reflecting blood–CSF barrier dysfunction and CSF lactate (*r* = 0.73–0.82), less so with CSF cell count, and not with blood CRP. In contrast to the elevated phosphatidylcholine concentrations in CSF, serum concentrations remained relatively unchanged. Taken together, these results suggest that increased free CSF phosphatidylcholines are sensitive biomarkers for bacterial meningitis and do not merely reflect inflammation but are associated with local disease and a shift in CNS metabolism.

## 1. Introduction

Despite all advances in diagnosis and treatment, bacterial meningitis continues to feature high morbidity and mortality. Untreated, it can be fatal in 50% of cases, and even when diagnosed early and treated adequately, 8–15% of the patients die, typically within 48 h of symptom onset. Furthermore, 10–20% of the survivors develop permanent sequelae including hearing loss and cognitive dysfunction [1]. In two large case series from the 1990s, for example, the case-fatality rate for adults with bacterial meningitis was approximately 25%, and transient or permanent neurologic morbidity occurred in 21–28% of survivors [2,3]. This is quite different in viral CNS infections: neuroinflammation tends to be less pronounced, many cases do not require hospitalization, severe disease occurs much less frequently, and serious sequelae are less common [4,5,6]. The timely diagnosis of bacterial meningitis is imperative not only to institute antibiotic treatment as soon as possible [1], but also to arrange for other indicated diagnostic and therapeutic measures in a timely manner, and to avoid unnecessary use of antiviral or antifungal drugs [7,8,9]. In spite of advances in microbial diagnostics, the causative pathogen is still not identified in all patients. For instance, it is possible to detect the pathogen in cerebrospinal fluid (CSF) in 70–90%. In blood culture the yield is decreased to 50–80% and further decreases by 20% after antibiotic pretreatment [10]. Moreover, the turnaround time to reporting results varies and may take several days depending on the nature of the assay and its availability in the respective health care setting. In particular, modern-day pathogen-directed diagnostics usually require technical equipment such as PCR or next-generation sequencing, which may not be available in less resource-rich settings. Therefore, there continues to be a great need for molecular biomarkers that reflect patterns in the host response to a given pathogen or class of pathogens and can potentially be measured with less sophisticated methods such as lateral diffusion assays. Even though metabolite profiling is usually carried out by mass spectrometry, it is often possible to develop simpler assays once a biomarker candidate has been validated.

Considering the high risk of neuronal damage and long-term sequelae in bacterial meningitis, there are efforts to expand the spectrum of treatments with anti-inflammatory and neuroprotective therapies [11,12]. Therefore, another reason for molecular profiling of patient CSF samples is to improve our understanding of pathophysiological networks and mechanisms and to identify disease-specific pathways that could serve as targets for host-directed treatments to reduce end-organ damage and, thus, improve clinical outcome.

Phosphatidylcholines are common constituents of cell membranes and also occur in free form in body fluids including blood and CSF. They are important substrates of phospholipases and other enzymes that can release fatty acid moieties such as arachidonic acid and linoleic acid, which can be catabolized further to yield a variety of lipid modulators of inflammation. In the brain, they play additional important roles in intraneuronal signal transduction and in regulating local levels of the neurotransmitter acetylcholine [13]. Inhibition of phosphatidylcholine synthesis by pathogenic bacteria leads to apoptosis of neurons [14] and, indeed, it has been suggested that phosphatidylcholines contribute to the balance between cell survival and death [15]. Elevated free phosphatidylcholine levels in CSF can be a sign of compromised membrane integrity, as occurs during neuronal damage, and removal of membrane fragments is one mechanism by which phagocytes in CNS may limit meningitis-associated inflammation and further cell death [16]. Thus, this class of phospholipids has potential as a source of biomarkers for CNS disorders characterized by intrinsic degeneration or extrinsic destruction, as is seen in bacterial meningitis [12]. 

We have previously assembled a cohort of 221 patients with bacterial, viral, autoimmune, and noninflammatory neuropsychiatric disorders and measured CSF concentrations of 188 small molecules using a targeted metabolomic approach based on mass spectrometry. By analyzing subsets of the data, we identified highly accurate biomarkers for CNS involvement in varicella-zoster virus (VZV) reactivation [17], for the identification of enterovirus meningitis in patients without pleocytosis [18], to distinguish between infectious and autoimmune neuroinflammation [19], and we have begun to identify biomarkers for bacterial meningitis [20]. We have now expanded the analysis of this cohort to screen for biomarkers that can accurately distinguish between bacterial and viral CNS infections and may reveal pathophysiological mechanisms that are preferentially active in bacterial meningitis. 

## 2. Methods

### 2.1. Study Cohort

This analysis is based on part of a cohort of CSF samples from 221 patients, which were collected between 2005 and 2013. Other aspects of this cohort have been described in [17,18,19,20]. The study was approved by the Ethics Committee of Hannover Medical School (file no. 2413–2014). For the current analysis, we analyzed CSF samples (*N* = 132) from patients with bacterial meningitis (BacM, *n* = 32), viral meningitis or encephalitis (*n* = 34), consisting of herpes simplex virus encephalitis (HSE, *n* = 9), varicella zoster virus meningoencephalitis (VZV ME, *n* = 15), enterovirus meningitis (EntM, *n* = 10), and noninflamed controls (*n* = 66) comprising normal pressure hydrocephalus (*n* = 35), Bell’s palsy (*n* = 11), and Gilles de la Tourette syndrome (*n* = 20). Serum samples, which were obtained at the time of lumbar puncture, were available from a subset of participants (bacterial meningitis *n* = 8, viral meningitis/encephalitis *n* = 13, controls *n* = 21). Diagnostic criteria (case definitions) are summarized in Table S1.

### 2.2. Standard Clinical Diagnostic Parameters

The following parameters were determined after lumbar puncture: CSF cell count, CSF protein concentration, CSF lactate concentration, Q-albumin ratio (CSF albumin/serum albumin), and IgG index (IgG ratio/Q-albumin ratio). Blood–CSF Barrier (BCB) dysfunction was scored from 0 (no dysfunction) to 3 (severe dysfunction), using age-corrected Q-albumin as described in [21]. Peripheral blood leukocyte counts and C-reactive protein (CRP) levels were determined in blood samples obtained at the time of lumbar puncture. Further details are given in [17,18,19].

### 2.3. Targeted Metabolomics

CSF metabolite concentrations were measured on a triple-quadrupole mass spectrometer (API4000, Sciex, Framingham, MA, USA) with an electrospray-ionization ion source coupled to a high-performance liquid chromatography system (SIL-HTc, Shimadzu, Japan) and the AbsoluteIDQ™ p180 kit and MetIDQ™ software (Biocrates Life Sciences, Innsbruck, Austria), as described in detail in [17]. Serum concentrations of phosphatidylcholines were measured on an AB SCIEX 5500 QTrap™ mass spectrometer (AB SCIEX, Darmstadt, Germany) using the MxP™ Quant 500 kit (Biocrates), following the manufacturer’s protocols (https://biocrates.com/mxp-quant-500-kit, accessed on 15 December 2020). Details about internal standards and quality controls (QC) are given in the manufacturer’s application note [22]. The Quant 500 kit measures the same glycerophospholipids as the p180 kit and is downward compatible with it, ensuring comparability of the data. 

### 2.4. Quality Screen

Since our previous studies have shown that CSF metabolite biomarkers are usually more highly concentrated in the more inflamed diagnostic group, we only included analytes that were detected above limit of detection (LOD = 3x the signal obtained with the blank in most analytes) in at least 80% of the bacterial meningitis samples, whereas concentrations in the comparator groups were not part of inclusion criteria. One analyte (PC.ae.C42.4) had to be removed from the analysis due to erratic values likely resulting from a technical error. Based on this screen, we included 100 analytes in the subsequent analyses, comprising 54 phosphatidylcholines (PCs), 5 lysophosphatidylcholines (lysoPCs), 12 sphingomyelins (SMs), 23 amino acids (AAs) and amino acid metabolites (AAMs), 5 acylcarnitines (ACs), and the sum of hexoses (Figure S1). All concentrations measured as <LOD were replaced with the value LOD/2.

### 2.5. Statistical Analysis

As the data were non-normally distributed, the Spearman method was used to assess correlations and the Mann–Whitney U and Kruskall–Wallis tests were used to compare differences in median values between groups and across groups, respectively. The Chi-squared test was used to assess differences in categorical parameters. For principal component analysis (PCA), data were log-transformed and analyzed using R package “FactoMineR” [23] and the PCA figure was visualized using R package “factoextra” [24]. Receiver operating characteristic (ROC) curve analysis was carried out using R package “ROCR” [25]. This procedure was repeated with 1000 bootstrap samples in order to compute a confidence interval (CI) for the area under the curve (AUC). Candidate biomarkers were then subjected to internal cross-validation using leave-one-out (jackknife) cross-validation [26]. Here, we applied both a simple cross-validation without the inner loop and a double cross-validation (including the inner loop) [27,28], whereby the double cross-validation featured 5 repeats of a 10-fold cross-validation followed by 1000 bootstraps. We calculated how many times a feature was selected in all iterations of the cross-validation within the subset of the 10 biomarker candidates with the highest AUC, using the frequency of selection as a measure of robustness of a biomarker. High AUC Abundance (HAUCA) curves were constructed as described previously [29,30]. Sensitivity, specificity, positive predictive value (PPV), negative predictive value (NPV) and cut-off values were calculated using the Youden index method [31].

## 3. Results

### 3.1. Study Cohort

Clinical and sociodemographic data revealed the differences expected from the natural history of the disease entities, including pronounced leukocytosis and elevated blood CRP in peripheral blood, and elevated CSF cell count, lactate levels (Table 1), and disruption of the BCB in bacterial meningitis (Table S2, which contains a complete list of all standard clinical parameters). On the other hand, there were no differences in age or sex between the bacterial meningitis, viral CNS infections, and control groups. Pathogenic bacteria isolated from the bacterial meningitis patients are summarized in Table S3. 

### 3.2. General Differences in CSF Metabolite Populations between Bacterial and Viral CNS Infections

**Figure 1:**
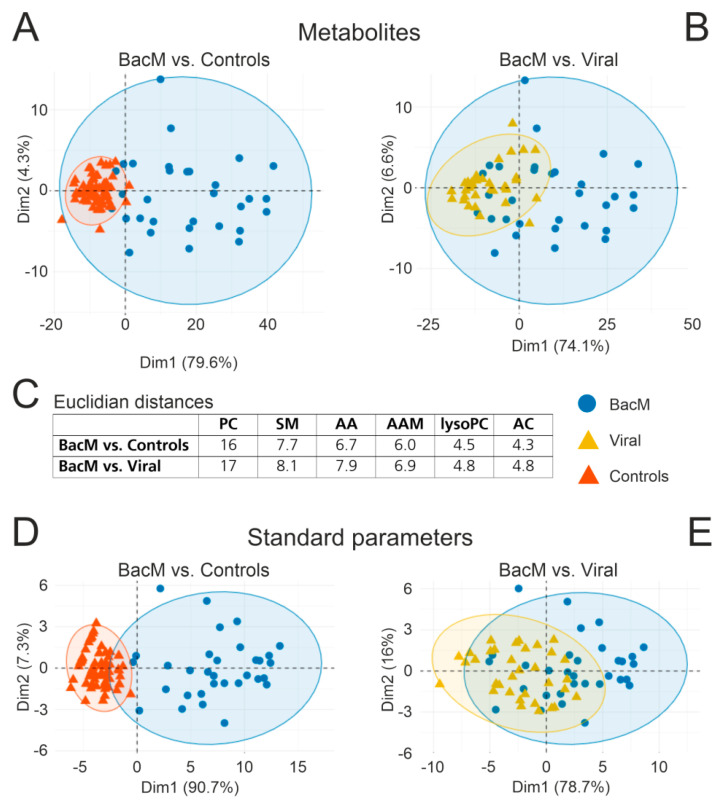
Differences in CSF metabolite populations between bacterial meningitis, viral CNS infections, and noninflamed controls are driven by changes in phosphatidylcholine concentrations. PCA was performed on the basis of 99 CSF analytes (54 PC, 5 lysoPC, 12 sphingomyelins, 23 amino acids and amino acid metabolites, 5 acylcarnitines) and 7 standard diagnostic CSF parameters (leukocyte count, lactate, protein, BCB disruption, Q albumin, IgG Index, Q IgG). (**A**) Bacterial meningitis vs. controls (metabolites). (**B**) Bacterial meningitis vs. viral CNS infections (metabolites). (**C**) Mean Euclidian distances (reflecting global changes in individual metabolite classes) between bacterial meningitis and controls or viral CNS infections. (**D**) Bacterial meningitis vs. controls (standard parameters). (**E**) Bacterial meningitis vs. viral CNS infections (standard parameters). Abbreviations: AA, amino acids; AAM, amino acid metabolites; AC, acylcarnitines; lysoPC, lysophosphatidylcholines; PC, phosphatidylcholines; SM, sphingomyelins. BacM, bacterial meningitis; Viral, viral CNS infection.

A principle component analysis revealed a clear separation of bacterial meningitis from the control samples and a somewhat less pronounced separation from the viral CNS infections (Figure 1A,B). Of note, the contribution to variance in the first dimension was much greater than in the second dimension and was almost exclusively due to changes in glycerophospholipids and sphingomyelins, whereas the second dimension was dominated by amino acid metabolism (Figure S2). A comparable degree of separation was also achieved on the basis of the standard CSF diagnostic parameters (Figure 1D,E).

Analysis of mean Euclidian distances confirmed that CSF metabolite reprogramming in bacterial meningitis vs. controls or viral CNS infections was by far greatest in phosphatidylcholines and least in lysophosphatidylcholines and acylcarnitines (Figure 1C). Thus, the CSF metabolite changes due to acute bacterial meningitis preferentially affected glycerophospholipid homeostasis.

### 3.3. Biomarker Screening and Internal Validation

A hierarchical clustering analysis showed a clade (node marked 1) with broad upregulation of glycerophospho- and sphingolipids in bacterial meningitis but, to a lesser extent, also some viral samples (mostly HSV encephalitis and VZV meningoencephalitis) (Figure S3). Two smaller clades (nodes 2 and 3) were identified consisting mostly of amino acids and their metabolites and acylcarnitines. Separation of the diagnostic groups was not perfect: while there was a bacterial meningitis clade containing 20 bacterial meningitis samples with highly upregulated phospholipids (node 4), there was also some coclustering with viral infections, especially HSV encephalitis. On the less inflamed end of the spectrum, there were still some viral samples within the clade predominantly comprised of control subjects (node 5). These results supported the above notion that changes in CSF phospholipid populations are a salient feature of bacterial meningitis, but that they are also found in viral CNS infections, albeit less pronounced. 

**Figure 2:**
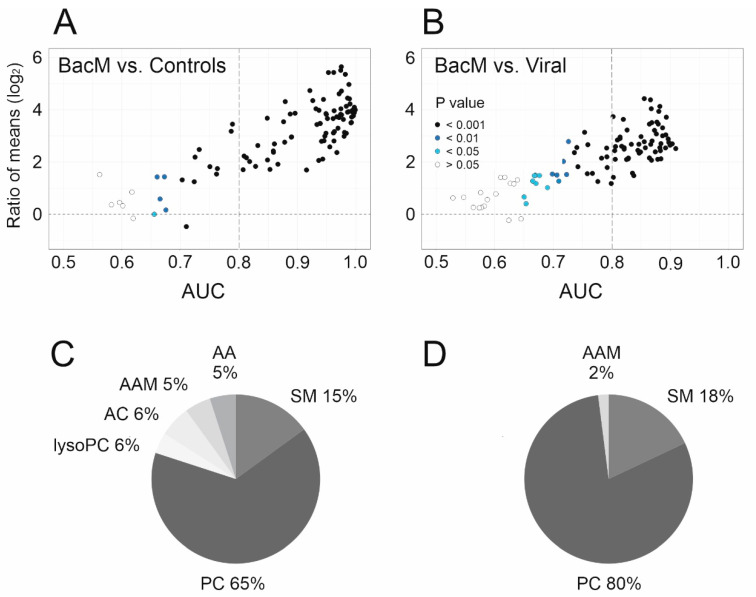
Phosphatidylcholines have the greatest biomarker potential to distinguish bacterial meningitis from viral CNS infections. (**A**,**B**). Dispersion plots based on binary receiver operating characteristic (ROC) curve analysis of the same 100 CSF metabolites as used for Figure 1. For each pair-wise comparison, ratio of mean concentrations (“fold change”, Bacterial meningitis/Controls and Bacterial meningitis/Viral CNS Infections) is plotted on the y-axis, area under the ROC curve (AUC) along the x-axis; each circle represents one metabolite, with the fill color indicating asymptotic significance of the ROC curve. The y-axis label of A also applies to B. (**A**) Bacterial meningitis/Controls. (**B**) Bacterial meningitis/Viral CNS infections. (**C**,**D**). Percentages of all biomarkers (AUC > 0.8, *p* < 0.05, lower CI > 0.5) that originate from each of the 6 metabolite subclasses. (**C**) Bacterial meningitis/Controls; (**D**) Bacterial meningitis/Viral CNS infections.

We then applied ROC curve analysis to screen the metabolite data set for biomarker candidates (Figure 2). In the comparison bacterial meningitis vs. controls, major differences in metabolite concentrations were apparent, which were mostly due to higher concentrations in bacterial meningitis, were highly significant, and yielded many (*n* = 79) biomarkers, defined as having an AUC ≥ 0.8, a lower CI not crossing below the chance line of 0.5, and an asymptotic *p* value of <0.05. Of note, even though the overall differences appeared to be smaller, 57 biomarkers were identified for the clinically relevant differentiation between bacterial meningitis and viral CNS infections (Figure 2A,B). We then used HAUCA curve analysis to assess the likelihood of finding false positives among these biomarkers. It turned out to be extremely low, as the upper 95% CI of AUC expected in a random data set was 0 for AUC ≥ 0.8 in both binary comparisons (Figure S4). When comparing biomarker potential for the distinction bacterial meningitis vs. viral CNS infections across the metabolite subclasses, phosphatidylcholines clearly possessed the greatest discriminatory potential. All 54 members of this class were significantly (*p* = 1.2 × 10^−25^–1.5 × 10^−4^) elevated in bacterial meningitis vs. controls, and 53/54 (*p* = 3.2 × 10^−10^–3.5 × 10^−3^) in bacterial meningitis vs. viral CNS infections. Indeed, they constituted 51 of the 79 (65%) total biomarker candidates for bacterial meningitis vs. controls, and 46/57 (80%) for the distinction from viral CNS infections (Figure 2C,D). Phosphatidylcholines also constituted the metabolite subclass with the greatest percentage in a subclass satisfying the above criteria for biomarker candidates for bacterial meningitis vs. viral CNS infections (Table S4). 

In the absence of an independent sample set that could be used for external validation, we performed internal cross-validation using the jackknife method. In this analysis, both the simple and double cross-validations selected the same phosphatidylcholines as the 10 most robust biomarkers (Table 2). All 10 exhibited accurate classification as evidenced by AUCs of 0.89–0.90. However, a parallel comparison with the standard CSF parameters revealed that lactate concentration had a slightly higher AUC and across the board had higher specificity and positive predictive value than the phosphatidylcholines (Table 3). On the other hand, four of the five top phosphatidylcholines had higher sensitivity and negative predictive values than lactate. Of note, despite its weak correlation with phosphatidylcholines, blood CRP also exhibited high sensitivity and negative predictive value.

### 3.4. Correlations with Parameters of Systemic and CNS Inflammation

**Figure 3:**
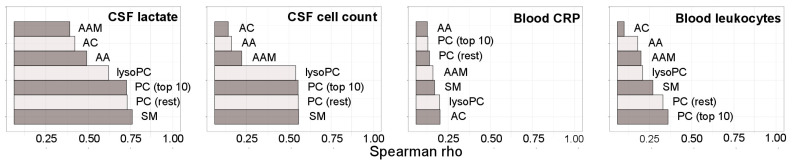
Correlations of the CSF metabolites in bacterial meningitis with CSF and blood parameters. Correlations (mean Pearson correlation coefficient) in 33 bacterial meningitis samples of the indicated metabolite classes with CSF lactate, CSF cell count, blood CRP, and blood leukocytes. Correlations with the remaining parameters are shown in Figure S5. Abbreviations: AA, amino acids; AAM, amino acid metabolites; AC, acylcarnitines; lysoPC, lysophosphatidylcholines; PC, phosphatidylcholines; SM, sphingomyelins.

We then tested to what extent the observed changes in bacterial meningitis in the various CSF metabolite groups correlated with parameters of local (measured in CSF) and systemic (measured in blood) inflammation (Figure 3 and Figure S5). The strongest correlation across all 100 metabolites was identified with Q-albumin (mean |*r*| = 0.72), closely followed by Q-IgG, protein, BCB dysfunction, all of which reflect dysfunction of the BCB, whereas increased protein can also be a sign of cell death. The correlation with CSF lactate was similar, whereas correlation with CSF cell count was only moderate, and those with blood leukocyte count and CRP were weak. In terms of metabolite subgroups, phosphatidylcholines and sphingomyelins exhibited the strongest correlations, again correlating most strongly with parameters reflecting BCB dysfunction and lactate, much less with cell count and only minimally with blood parameters. Of note, the only significant (*p* ≤ 0.05) negative correlations were those of hexoses (in CSF mostly represented by glucose) with lactate and cell count. Taken together, these results strongly suggest that the observed changes in overall metabolite populations and in the phosphatidylcholine biomarkers (i) reflect local CNS disease more than systemic manifestations and (ii) are most closely associated with disruption of the BCB, cell death, and a disease-associated shift in central carbon metabolism to glycolysis. 

### 3.5. Differentiation between Bacterial Etiologies

We then tested the hypothesis that concentrations of the phosphatidylcholine biomarkers could differentiate between infection with typical meningitis pathogens such as pneumo- and meningococci (which may cause aggressive disease in immunocompetent individuals) and infection with opportunistic pathogens, which are more likely to be pathogenic in immunocompromised or multimorbid individuals. Dividing the bacterial meningitis samples into two groups, typical and opportunistic meningitis pathogens (see classification marked in Table S3), indeed demonstrated that concentrations of 8 of the 10 most accurate biomarkers (all of which were phosphatidylcholines) were significantly higher in patients with a classic meningitis pathogen as etiology (*p* = 0.009–0.048), whereas lactate (*p* = 0.08) and leukocyte count (*p* = 0.55) did not achieve this degree of significance (Figure S6). 

### 3.6. Increased Phosphatidylcholine Concentrations in CSF but Not Serum

**Figure 4:**
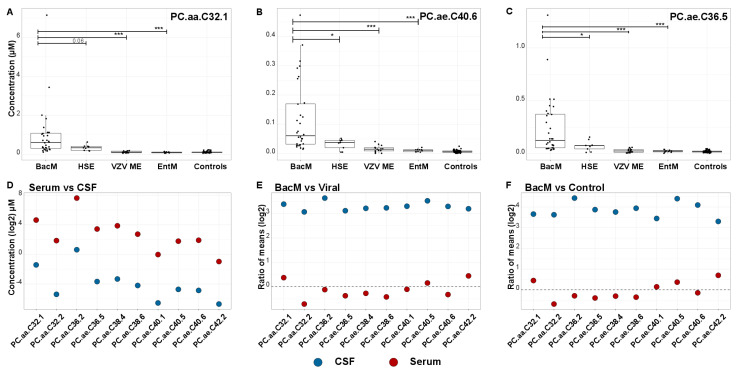
Concentrations of phosphatidylcholine biomarkers in bacterial and viral CNS infections and noninflamed controls. (**A**–**C**). CSF concentrations of the three best validated markers in bacterial meningitis, viral CNS infections (all), HSV encephalitis, VZV meningoencephalitis, enterovirus meningitis, and controls. (**A**) PC.aa.C32.1. (**B**) PC.ae.C40.6. (**C**) PC.ae.C36.5. (**D**–**F**). Comparison of CSF vs. serum concentrations of the 10 most robust metabolite biomarkers in the eight bacterial meningitis cases in which paired CSF-serum samples were available. (**D**) Mean CSF (blue) and serum (brown) concentrations. (**E**,**F**). Ratios of mean concentrations of the 10 phosphatidylcholine biomarkers in bacterial meningitis/controls (**E**) and bacterial meningitis/viral CNS infections (**F**) in CSF (blue) and serum (brown). * *p* ≤ 0.05, *** *p* ≤ 0.001.

Figure 4 shows CSF concentrations of the top three markers in bacterial meningitis, viral CNS infections according to each of the three causative pathogens, and controls. Concentrations were clearly the highest in bacterial meningitis, and among the three viral etiologies they were highest in HSV encephalitis, which is associated with the most deleterious effects on CNS tissue and the poorest clinical outcome. A similar trend was observed in the other phosphatidylcholine biomarkers. To test whether concentrations of these markers were regulated in a similar fashion systemically (i.e., outside the CNS), we measured their concentrations in serum samples, which were available from 8 patients with bacterial meningitis, 13 with viral CNS infections, and 21 controls. As expected, concentrations were substantially higher in serum than in CSF [32] (Figure 4D), but the marked upregulation in bacterial meningitis CSF was not observed in serum. On the contrary, there appeared to be a trend towards lower serum concentrations in bacterial meningitis, especially when compared to viral CNS infections (Figure 4E,F), but none of the differences were significant (lowest *p* = 0.28, Mann–Whitney U test, PC.aa.C32.1). 

## 4. Discussion

We performed a targeted metabolomic analysis of CSF samples from patients with bacterial meningitis, viral CNS infections and noninflammatory controls, and identified free phosphatidylcholines as the molecular class with the highest biomarker potential, in particular to increase sensitivity and negative predictive value for a diagnosis of bacterial meningitis, and to distinguish between meningitis due to typical vs. opportunistic bacterial pathogens. 

We previously found that increased phosphatidylcholines were among the most accurate CSF biomarkers to detect CNS involvement in VZV reactivation [17] and enterovirus meningitis in patients without CSF pleocytosis [18]. Thus, increased release of phosphatidylcholines into CSF is not a unique feature of bacterial infections. Our current study confirmed that some phosphatidylcholines are also raised in viral CNS infections, and their increase correlates with the degree of parenchymal involvement, as levels of the detected phosphatidylcholine biomarkers increased from enterovirus meningitis over VZV meningoencephalitis to HSV encephalitis, which confers the greatest risk of parenchymal damage and long-term sequelae [6]. Thus, there may be some overlap in pathophysiological involvement of membrane phospholipids in bacterial meningitis and the more severe forms of viral CNS infections. However, median levels measured in bacterial meningitis were considerably higher, likely reflecting the more pronounced cell damage in this disease entity. In order to approach the association of the identified phosphatidylcholines with bacterial meningitis from a different angle, we tested how much overlap there is between the 10 most accurate phosphatidylcholine biomarkers identified in the present study and the most robust biomarkers for the above-mentioned viral infections. Underscoring the differences between phosphatidylcholine reprogramming in bacterial vs. viral CNS infections, only one of the top 10 biomarkers from the current study (PC.ae.C36.5) had also been identified as a biomarker for a viral CNS infection. Firstly, this specificity strongly argues against the notion that the increased CSF phosphatidylcholine levels in bacterial meningitis merely resulted from nonspecific immigration through a leaky blood–CSF barrier. Secondly, PC.ae.C36.5 was a moderately accurate biomarker (AUC = 0.821) to differentiate enterovirus meningitis with normal CSF cell count from noninflamed control samples, which may be due to its ability to reflect release of cell membrane constituents despite the absence of overt neuroinflammation [18]. Very little is known about cell type-specific distribution of glycerophospholipids in CNS. However, it is tempting to speculate that the pattern of phosphatidylcholines in CSF may reflect not only the extent of parenchymal damage but also involvement of specific cell types. 

From the clinical point of view, it is notable that CSF lactate turned out to be the metabolite biomarker with the overall greatest accuracy, thus further validating lactate as a routine CSF biomarker to differentiate between bacterial and aseptic meningitis [33]. Even though the phosphatidylcholine biomarkers fell slightly short of lactate in terms of AUC, several had higher sensitivity and negative predictive values—i.e., properties that are important when it is critical (as in bacterial meningitis) to minimize the frequency of false negative test results that would lead to undertreatment. It is of pathogenetic relevance that the phosphatidylcholine biomarkers were superior to lactate or cell count to differentiate between meningitis caused by typical meningitis pathogens such as pneumococci or by uncommon (opportunistic) pathogens such as nonaureus staphylococci. This phenomenon is likely explained by the notion that such opportunistic infections can cause considerable neuroinflammation but that cell damage is often less severe. These findings also agree well with our previous report that PC.ae.44:6 is a sensitive biomarker to distinguish bacterial meningitis from the combined groups of viral CNS infections and noninfectious neuroinflammatory disorders, and that its concentrations were also lower in samples from patients with opportunistic bacterial pathogens [20]. In our current study, PC.ae.44:6 had a lower AUC for the discrimination between bacterial meningitis and viral CNS infections (0.86) than the AUC reported in the previous study (0.93). This is explained by the fact that the distinction in that report was between bacterial meningitis and the larger combined group of viral CNS infections plus noninfectious neuroinflammatory disorders, which tended to be less inflamed. 

Our study is unique in that we were able to compare concentrations of the phosphatidylcholine biomarkers in paired CSF and serum samples. In peripheral blood, concentrations of many phosphatidylcholines decrease in systemic inflammatory disorders. This may be due to a combination of alterations in membrane signaling, catabolism by free phospholipases and other enzymes in blood, and removal in the reticuloendothelial system ([32] and references therein). In spite of the markedly increased levels of the top 10 phosphatidylcholine biomarkers in CSF, their concentrations did not change significantly in serum. This indicates that their alterations in CSF do not reflect systemic inflammation but result directly from local disease and, thus, sampling of CSF is the most promising approach to search for disease-associated alterations in phosphatidylcholine homeostasis in CNS infections. 

Even though our study features a sample size typical of a single-center cohort, the conclusions are limited as we could not perform an external validation. This is particularly true for the comparison “typical” vs. “opportunistic” pathogens. In addition, the exact molecular identity of some of the investigated phosphatidylcholines is uncertain, as the designated molecular species may actually correspond to up to six isobars or isomers with known chemical structures (Table S5). In addition, this targeted metabolomics approach was focused on a small number of metabolite classes, and pathogenetically plausible lipids such as ceramides were not included. The Human Metabolome database (https://hmdb.ca, accessed on 15 December 2020) lists 376 endogenous CSF metabolites, and a broader screening would likely detect additional and perhaps more accurate biomarkers. On the other hand, we used prospectively collected specimens that were processed according to unified protocols and had a definitive identification of bacterial/viral pathogens.

## 5. Conclusions and Outlook

Our results underscore the value of CSF lipid profiling for the discovery of biomarkers for CNS infections and show that raised phosphatidylcholine concentrations in CSF are not the result of systemic inflammation but reflect local disease activity, likely by measuring release from perturbed cell membranes. Measuring free phosphatidylcholines in CSF may provide added value to diagnosis and risk stratification of patients with a clinical suspicion of meningitis, specifically by providing high sensitivity and NPV, distinguishing between patients infected with classic vs. atypical meningitis pathogens, and identifying those at greatest risk of neuronal damage and clinical complications. Further research should focus on identifying the exact molecular species that define the discriminatory ability of the identified phosphatidylcholine biomarkers, validating their diagnostic performance in larger cohorts, assessing their prognostic value in longitudinal study designs, and ultimately developing simpler assays that can be used as rapid diagnostics at the time of lumbar puncture. 

## Figures and Tables

**Table 1:** Demographic and selected clinical laboratory characteristics.

		Bacterial Meningitis	HSV Encephalitis	Enterovirus Meningitis	VZV Meningo- Encephalitis	Controls	
	Median (Range)						*p* Value
	Age (years)	51 (18–83)	56 (29–76)	39 (22–76)	51 (13–80)	50 (19–91)	0.98 ^a^
	Sex (female %)	47	22	50	35	43	0.5 ^b^
Blood	Leukocyte count (1000/µL)	16.2 (6.5–48.4)	10.1 (1.8–12.8)	7 (4–14)	6.9 (3.8–13.6)	6.8 (3.7–11.9)	3.3 × e^−11^ ^a^
	C-reactive protein (mg/L)	100 (1–429)	3 (1–102)	4 (1–39)	2 (1–25)	1.5 (0.3–31)	8.6 × e^−13^ ^a^
CSF	Cell count (1/µL)	764.9 (4.3–18,800)	90.7 (16–723)	9.2 (0.7–619)	60.5 (1.7–1536)	1 (0.3–11.3)	2.2 × e^−16^ ^a^
	Lactate (mmol/L)	7.2 (1.8–21.9)	2.3 (1.8–3.4)	1.8 (1.6–3.6)	2.7 (1.5–5.5)	1.6 (1.2–2.5)	2.2 × e^−16^ ^a^

^a^ Kruskal–Wallis test ^b^ Chi—squared test. HSV = herpes simplex virus; VZV = varicella zoster virus.

**Table 2:** Comparison of standard parameters and the 10 most robust CSF metabolite biomarkers to differentiate between bacterial meningitis and viral CNS infections.

Standard Parameters			CSF Metabolites			
Parameter	AUC (95% CI)	Ratio of Means	Metabolite ^a^	AUC (95% CI)	Ratio of Means	Selection Frequency (Non-Nested/Nested) ^b^
CSF lactate	0.93 *** (0.86–0.99)	3.5	PC.aa.C32.1	0.91 *** (0.84–0.97)	5.70	1/1
Blood CRP	0.9 1 *** (0.83–0.97)	11.4	PC.ae.C40.6	0.90 *** (0.83–0.97)	6.47	1/1
Blood leukocytes	0.85 *** (0.79–0.94)	3.3	PC.ae.C36.5	0.90 *** (0.83–0.96)	6.24	1/1
CSF cell count	0.82 *** (0.70–0.91)	21.7	PC.ae.C38.4	0.90 *** (0.82–0.96)	6.73	1/1
CSF Protein	0.81 *** (0.71–0.90)	3.4	PC.aa.C32.2	0.90 *** (0.82–0.96)	7.35	1/0.9
Q albumin	0.80 *** (0.65–0.88)	3.3	PC.ae.C40.5	0.89 *** (0.81–0.96)	13.12	0.98/0.6
Q IgG	0.77 *** (0.83–0.97)	3.2	PC.ae.C40.1	0.89 *** (0.80–0.96)	8.21	0.94/0.7
BCB disruption	0.722 ** (0.60–0.84)	n/a	PC.ae.C38.6	0.89 *** (0.81–0.96)	5.80	0.98/0.8
IgG-index	0.621 (0.51–0.77)	0.99	PC.aa.C36.2	0.89 *** (0.80–0.95)	9.89	0.86/0.5
			PC.ae.C42.2	0.89 *** (0.80–0.96)	8.01	0.70/0.5

**^a^** A list of the corresponding molecular species and their Lipidmaps IDs is shown in Table S5. ^b^ Frequency of selection in leave-one-out (jackknife) cross-validation, using either the unnested or nested procedure. The same 10 markers were selected in the two procedures. ** *p* ≤ 0.01, *** *p* ≤ 0.001.

**Table 3:** Comparison of diagnostic parameters between CSF lactate, CSF cell count, blood CRP, and the five most accurate phosphatidylcholine biomarkers.

Biomarkers	Sensitivity	Specificity	PPV	NPV	Cut-Off Value
CSF_lactate	0.88	0.91	0.90	0.88	3.9 mmol/L
CSF cell count	0.75	0.79	0.77	0.77	240 cells/µL
Blood CRP	0.91	0.85	0.85	0.9	24 mg/L
PC.aa.C32.1	0.91	0.82	0.83	0.90	0.22 µM
PC.ae.C40.6	0.91	0.74	0.76	0.89	0.02 µM
PC.ae.C36.5	0.91	0.74	0.76	0.89	0.05 µM
PC.ae.C38.4	0.75	0.85	0.83	0.78	0.11 µM
PC.aa.C32.2	0.94	0.68	0.73	0.92	0.01 µM

PPV, positive predictive value; NPV, negative predictive value.

## Data Availability

The data used and/or analyzed are available at the data cloud of the TWINCORE Centre for Experimental and Clinical Infection Research through the following link: http://81.14.181.117:8080/share.cgi?ssid=0cM1j5f.

## Supplementary Materials

The following are available online at https://www.mdpi.com/article/10.3390/cells10051115/s1, Figure S1: Quality screen used to identify analytes to be included in the analysis. Figure S2a: Scree plot illustrating the contribution of each principle component to overall variance in 100 metabolites in the PCA shown in Figure 1B. Figure S2b: Contributions of individual metabolites to variance in the PCA shown in Figure 1 and the Scree Plot shown in Figure 2A. Figure S3. Unsupervised hierarchical biclustering analysis using the same metabolite data as for Figure 1. Figure S4. HAUCA curve analysis to assess the likelihood of false positive biomarker identification. Figure S5. Correlations of metabolite subgroups with the CSF and blood parameters not shown in Figure 3. Figure S6. Phosphatidylcholine levels are lower in meningitis due to opportunistic vs. typical meningitis bacterial pathogens. Table S1: Diagnostic criteria and additional clinical information. Table S2: Demographic and clinical laboratory characteristics. Table S3: Causative pathogens in 32 patients with bacterial meningitis. Table S4a: Differences among the metabolite classes in biomarker potential to differentiate bacterial meningitis from non-inflamed controls. Table S4b: Differences among the metabolite classes in biomarker potential to differentiate bacterial meningitis from non-inflamed controls. Table S5: Top 10 biomarkers: list of the corresponding molecular species and their Lipidmaps ID.
