# Supplementary material for: Elevated Free Phosphatidylcholine Levels in Cerebrospinal Fluid Distinguish Bacterial from Viral CNS Infections
Source: Cells. 2021 May 6;10(5):1115. doi: 10.3390/cells10051115 (PMC8148106; doi:10.3390/cells10051115)
Supplement: Supplementary file 1 [file cells-10-01115-s001.zip › cells-1169182-supplementary.pdf]

**Supplementary Materials**

**Table S1.** Diagnostic criteria and additional clinical information. *

| Diagnosis | Criteria | Disease grade/activity | Systemic treatment at time of lumbar puncture [a] |
|---|---|---|---|
| Bacterial meningitis (n=32) | Clinical meningitis, pleocytosis (≥5 cells/µL), detection of bacterial pathogen in CSF or blood [b] | Detected pathogens: *See Table S3* | Antibiotics (n=4)<br>- ceftriaxone, ampicillin<br>- ciprofloxacin, metronidazole<br>- ceftriaxone, cephazolin<br>- gentamycin, ampicillin, ceftazidime |
| HSV encephalitis (n=9) | Mental status changes and positive HSV PCR or ASI >1.5 | | Acyclovir (n=4)<br>- with ampicillin (n=1) |
| VZV meningitis/encephalitis (n=15) | Detection of VZV in CSF by PCR and/or intrathecal synthesis of VZV IgG,<br>clinical meningitis/encephalitis with or without typical zoster rash | Meningitis, 73%<br>Encephalitis, 27% | Immunosuppression (n=1)<br>-rituximab, bendamustin<br>(3weeks prior to lumbar puncture due to mantle cell lymphoma) |
| Enterovirus meningitis (n=10) | Clinical meningitis and detection of enterovirus in CSF by PCR | Acute onset (100%) (symptoms ≤3 months) | - |
| Tourette syndrome (n=20) | Criteria according to DSM-5 [c] | Classified by YGTSS-TTS<br>mild (10%)<br>moderate (70%)<br>severe (20%) | Symptomatic treatment (n=3)<br>-Abilify (n=1)<br>-Dronabinol (n=1)<br>-Sativex (n=1) |
| Bell's palsy (n=11) | Facial nerve palsy without evidence of infectious etiology or pleocytosis | | - |
| Normal pressure hydrocephalus (n=35) | Normal CSF pressure, typical findings on CT or MRI, at least one symptom of Hakim triad[d] | | - |

* Adapted from ref. 19 (Sühs et al. J Infect Dis 2019; 220:127-138) with kind permission by the publisher. [a] Excluding antipyretics, analgesics, and medications for unrelated conditions; [b] Brouwer, M.C, Thwaites, G.E., Tunkel A.R, van de Beek, D. Lancet. 380 (2012) 1684-1692. [c] American Psychiatric Association (2013) *Diagnostic and Statistical Manual of Mental Disorders* (5th ed.) [d] Hakim et al. N Engl J Med, 1965; 2:307-27; Abbreviation: YGTSS-TTS = Yale Global Tic Severity Scale Total Tic Score.

**Table S2.** Demographic and clinical laboratory characteristics.

| | Parameter | Bacterial meningitis | HSV encephalitis | Enterovirus meningitis | VZV meningo-encephalitis | Controls | |
|---|---|---|---|---|---|---|---|
| | | Median (Range) | | | | | P value |
| | Age (years) | 51 (18 - 83) | 56 (29 – 76) | 39 (22 – 76) | 51 (13 – 80) | 50 (19 – 91) | 0.98 [a] |
| | Female % | 47 | 22 | 50 | 35 | 43 | 0.5 [b] |
| **Blood** | Leukocyte count (1000/µL) | 16.2 (6.5 - 48.4) | 10.1 (1.8 - 12.8) | 7 (4 - 14) | 6.9 (3.8 - 13.6) | 6.8 (3.7 - 11.9) | 3.3e-11 [a] |
| | C-reactive protein (mg/L) | 100 (1 - 429) | 3 (1 - 102) | 4 (1 - 39) | 2 (1 - 25) | 1.5 (0.3 - 31) | 8.6e-13 [a] |
| **CSF** | Cell count (1/µl) | 764.9 (4.3 - 18800) | 90.7 (16 - 723) | 9.2 (0.7 - 619) | 60.5 (1.7 - 1536) | 1 (0.3 - 11.3) | 2.2e-16 [a] |
| | Lactate (mmol/L) | 7.2 (1.8 - 21.9) | 2.3 (1.8 - 3.4) | 1.8 (1.6 - 3.6) | 2.7 (1.5 - 5.5) | 1.6 (1.2 - 2.5) | 2.2e-16 [a] |
| | Protein (mg/L) | 2.1 (0.35 - 9.9) | 1.2 (0.5 - 2.3) | 0.5 (0.24 - 0.98) | 0.67 (0.42 - 2.1) | 0.4 (0.15 - 0.85) | 1e-15 [a] |
| | Q IgG | 25.3 (0.69 - 139.3) | 19.6 (4.5 - 58.4) | 5 (1.3 - 13.6) | 9.5 (3.2 - 21.1) | 2.7 (0.8 - 10.9) | 2.3e-15 [a] |
| | IgG Index | 0.65 (0.03 - 0.84) | 0.76 (0.5 - 1.3) | 0.51 (0.47 - 0 .63) | 0.56 (0.5 - 1.2) | 0.5 (0.4 - 1) | 7.3e-10 [a] |
| | Q albumin | 46.7 (3.2 - 176.9) | 31.8 (6.8 - 44.4) | 8.9 (2.7 - 21.7) | 12.4 (6.3 - 41.1) | 5.3 (1.5 - 15.7) | 4.2e-16 [a] |
| | BCB disruption (%) | | | | | | |
| | *None (1)* | 3 | 11 | 25 | 21 | 70 | 0.0005 [b] |
| | *Light (2)* | 19 | 11 | 62.5 | 36 | 29 | |
| | *Moderate (3)* | 19 | 11 | 12.5 | 29 | 1 | |
| | *Severe (4)* | 59 | 67 | 0 | 14 | 0 | |

[a] Kruskal - Wallis test [b] Chi – squared test. HSV = herpes simplex virus; VZV = varicella zoster virus.

**Table S3.** Causative pathogens in 32 patients with bacterial meningitis. [a]

| | **Pathogen** | n (%) |
|---|---|---|
| **Streptococci** | *S. pneumoniae* | 12 (36) |
| | *S. pyogenes* | 1 (3.0) |
| | *S. salivarius* [b] | 1 (3.0) |
| | *S. anginosus* [b] | 1 (3.0) |
| **Staphylococci** | *S. aureus* | 3 (9.1) |
| | *S. epidermidis* [b] | 2 (6.1) |
| | *S. hominis* [b] | 1 (3.0) |
| | *S. warneri* [b] | 1 (3.0) |
| **Others** | *N. meningitides* | 3 (9.1) |
| | *Listeria monocytogenes* | 3 (9.1) |
| | *H. influenzae* | 1 (3.0) |
| | *E. coli* | 1 (3.0) |
| | *Micrococcus luteus* [b] | 1 (3.0) |
| | *Peptostreptococcus* sp. [b] | 1 (3.0) |
| | *Bacillus* sp.[b] | 1 (3.0) |

[a]N = 33 isolates due to coinfection. Additional coinfections (second pathogens not included in the analysis): *S. pneumoniae* / HSV-2, *L. monocytogenes* / *Borrelia burgdorferi*. [b] Pathogen commonly considered opportunistic and treated as such in Fig. S5. Adapted from ref. 20, de Araujo L.S. et al. J Trans Med 18, 9 (2020) (copyright by the author, F. Pessler).

**Table S4a.** Differences among the metabolite classes in biomarker potential to differentiate bacterial meningitis from non-inflamed controls.

| Metabolite class | No. included in analysis | No. qualifying as biomarker [b] | % of all analytes | % of metabolite class |
|---|---|---|---|---|
| All | 100 | 79 | 79 | n/a |
| Phosphatidylcholines | 54 | 51 | 51 | 94 |
| Sphingomyelins | 12 | 12 | 12 | 100 |
| Acylcarnitines | 5 | 5 | 5 | 100 |
| Amino acids | 18 | 4 | 4 | 22 |
| Amino acid metabolites | 5 | 4 | 4 | 80 |
| Lysophosphatidylcholines | 5 | 3 | 3 | 60 |

**Table S4b.** Differences among the metabolite classes in biomarker potential to differentiate bacterial meningitis from viral CNS infections.

| Metabolite class | No. included in analysis | No. qualifying as biomarker[b] | % of all analytes | % of metabolite class |
|---|---|---|---|---|
| All | 100 | 57 | 57 | n/a |
| Phosphatidylcholines | 54 | 46 | 46 | 85 |
| Sphingomyelins | 12 | 10 | 10 | 83 |
| Amino acid metabolites | 5 | 1 | 1 | 20 |
| Lysophosphatidylcholines | 5 | 0 | 0 | 0 |
| Acylcarnitines | 5 | 0 | 0 | 0 |
| Amino acids | 18 | 0 | 0 | 0 |

[a]Analysis based on the ROC analysis presented in Fig 2. The metabolite classes are ranked according to "% of metabolite class" in descending order for the comparison bacterial meningitis vs. viral CNS infections. [b]AUC $\geq 0.8$, AUC 95% lower bound CI $\geq 0.5$, $p \leq 0.05$ for asymptotic significance of ROC curve.

**Table S5.** Top 10 biomarkers: list of the corresponding molecular species and their Lipidmaps ID (adapted from ref. 22).

| Short Name | Molecule | LMID |
|---|---|---|
| PC aa C32:1 | PC(14:0/18:1(11Z)) | LMGP01010490 |
| PC aa C32:1 | PC(14:0/18:1(9Z)) | LMGP01010492 |
| PC aa C32:1 | PC(16:0/16:1(9Z)) | LMGP01010566 |
| PC aa C32:1 | PC(18:1(9Z)/14:0) | LMGP01010882 |
| PC aa C32:1 | phosphatidylcholine 32:1 | |
| PC aa C32:2 | PC(14:0/18:2(9Z,12Z)) | LMGP01010496 |
| PC aa C32:2 | PC(16:1(9Z)/16:1(9Z)) | LMGP01010684 |
| PC aa C32:2 | phosphatidylcholine 32:2 | |
| PC aa C32:2 | PC aa C32:2 | |
| PC aa C36:2 | PC(16:0/20:2(11Z,14Z)) | LMGP01011469 |
| PC aa C36:2 | PC(18:0/18:2(9Z,12Z)) | LMGP01010768 |
| PC aa C36:2 | PC(18:1(11Z)/18:1(11Z)) | LMGP01010841 |
| PC aa C36:2 | PC(18:2(9Z,12Z)/18:0) | LMGP01010935 |
| PC aa C36:2 | phosphatidylcholine 36:2 | |
| PC aa C36:2 | 1,2-dioleoyl-sn-glycero-3-phosphocholine | LMGP01010890 |
| PC aa C36:2 | 1-octadecanoyl-2-[(10Z,12Z)-octadecadienoyl]-sn-glycero-3-phosphocholine | LMGP01010764 |
| PC aa C36:2 | 1-octadecanoyl-2-[(2E,4E)-octadecadienoyl]-sn-glycero-3-phosphocholine | LMGP01010765 |
| PC aa C36:2 | 1-octadecanoyl-2-[(6Z,9Z)-octadecadienoyl]-sn-glycero-3-phosphocholine | LMGP01010766 |
| PC aa C36:2 | 1,2-di-octadecenoyl-sn-glycero-3-phosphocholine | |
| PC aa C36:5 | PC(16:0/20:5(5Z,8Z,11Z,14Z,17Z)) | LMGP01010633 |
| PC aa C36:5 | PC(16:1(9Z)/20:4(5Z,8Z,11Z,14Z)) | LMGP01010695 |
| PC aa C36:5 | phosphatidylcholine 36:5 | |
| PC aa C36:5 | 1-[(7Z)-hexadecenoyl]-2-[(5Z,8Z,11Z,14Z)-eicosatetraenoyl]-sn-glycero-3-phosphocholine | LMGP01011305 |
| PC ae C36:5 | PC(P-16:0/20:4(5Z,8Z,11Z,14Z)) | LMGP01030010 |
| PC ae C36:5 | PC(o-16:1(9Z)/20:4(8Z,11Z,14Z,17Z)) | |
| PC ae C36:5 | phosphatidylcholine O-36:5 | |
| PC ae C36:5 | 1-hexadecyl-2-[(5Z,8Z,11Z,14Z,17Z)-eicosapentaenoyl]-sn-glycero-3-phosphocholine | LMGP01020058 |
| PC ae C38:4 | PC(o-18:0/20:4(8Z,11Z,14Z,17Z)) | LMGP01020102 |
| PC ae C38:4 | phosphatidylcholine O-38:4 | |
| PC ae C38:6 | PC(o-16:0/22:6(4Z,7Z,10Z,13Z,16Z,19Z)) | LMGP01020064 |
| PC ae C38:6 | phosphatidylcholine O-38:6 | |
| PC ae C40:1 | PC(o-18:1(9Z)/22:0) | LMGP01020268 |
| PC ae C40:1 | phosphatidylcholine O-40:1 | |
| PC ae C40:1 | 1-octadecyl-2-[(11Z)-docosenoyl]-sn-glycero-3-phosphocholine | LMGP01020213 |
| PC ae C40:5 | PC(o-20:1(11Z)/20:4(8Z,11Z,14Z,17Z)) | |
| PC ae C40:5 | phosphatidylcholine O-40:5 | |
| PC ae C40:5 | 1-octadecyl-2-[(4Z,7Z,10Z,13Z,16Z)-docosapentaenoyl]-sn-glycero-3-phosphocholine | LMGP01020107 |
| PC ae C40:6 | PC(o-18:0/22:6(4Z,7Z,10Z,13Z,16Z,19Z)) | LMGP01020110 |
| PC ae C40:6 | phosphatidylcholine O-40:6 | |
| PC ae C42:2 | PC(o-18:2(9Z,12Z)/24:0) | LMGP01020273 |
| PC ae C42:2 | phosphatidylcholine O-42:2 | |
| PC ae C42:2 | 1-icosyl-2-[(13Z,16Z)-docosadienoyl]-sn-glycero-3-phosphocholine | LMGP01020242 |

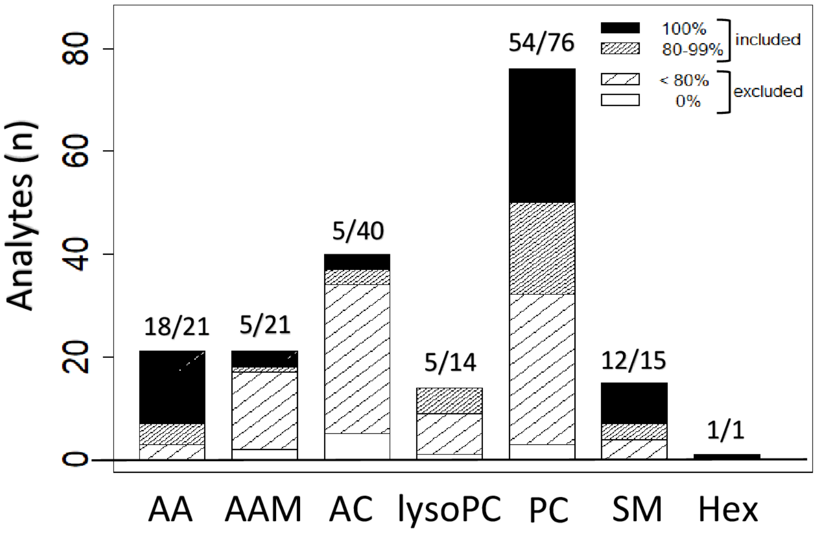


**Figure S1. Quality screen used to identify analytes to be included in the analysis.** Analytes were included that were detected ≥LOD in ≥80% of the bacterial meningitis samples. The numbers on top of the bars state the number of analytes that passed this screen divided by the total number of analytes in the respective metabolite subgroup. Detection efficiency in bacterial meningitis was highest for amino acids, but phosphatidylcholines constituted the largest group of included analytes. Abbreviations: AA, amino acids; AAM, amino acid metabolites; AC, acylcarnitines; lysoPC, lysophosphatidylcholines; PC, phosphatidylcholines; SM, sphingomyelins.

A

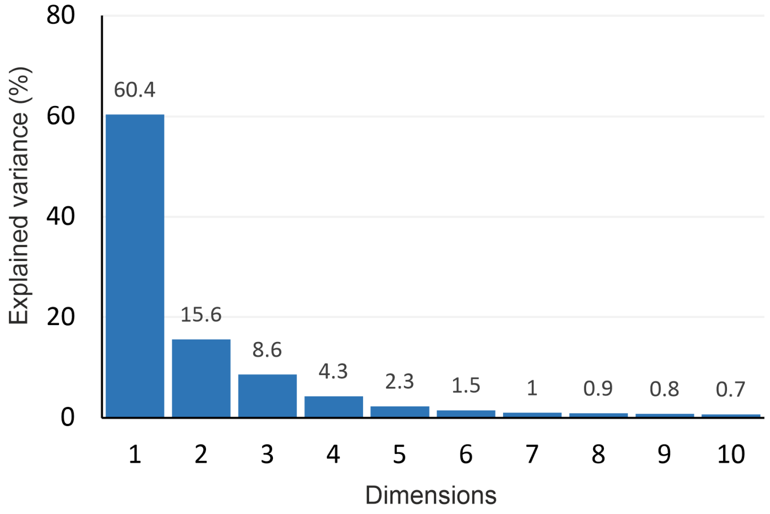


**Figure S2A.** Scree plot illustrating the contribution of each principle component to overall variance in 100 metabolites in the PCA shown in Fig. 1B.

**Figure S2B (Table).** Contributions of individual metabolites to variance in the PCA shown in Fig. 1 and the Scree Plot shown in Fig. 2A. *

| Dimension 1 | | Dimension 2 | |
|---|---|---|---|
| **Metabolites** | **Explained variance (%)** | **Metabolites** | **Explained variance (%)** |
| PC.ae.C34.0 | 1.58 | Asn | 5.23 |
| PC.ae.C36.2 | 1.57 | C4 | 5.17 |
| PC.ae.C40.6 | 1.55 | Gly | 5.12 |
| SM.C16.0 | 1.54 | C0 | 4.89 |
| PC.ae.C38.4 | 1.54 | t4.OH.Pro | 4.87 |
| PC.ae.C36.3 | 1.53 | C3 | 4.78 |
| SM.OH.C16.1 | 1.53 | His | 4.70 |
| SM.OH.C14.1 | 1.53 | Thr | 4.62 |
| PC.aa.C34.4 | 1.52 | Tyr | 4.46 |
| SM.C24.0 | 1.52 | C2 | 4.30 |
| PC.aa.C40.4 | 1.50 | Total DMA | 4.21 |
| SM.C16.1 | 1.49 | Leu | 4.17 |
| PC.aa.C36.1 | 1.49 | Ala | 4.13 |
| SM.OH.C22.2 | 1.49 | Ser | 4.05 |
| SM.C24.1 | 1.48 | Trp | 3.87 |

* Phospholipids make the greatest contributions to variance in a principle component analysis containing bacterial meningitis and viral meningitis/encephalitis. Contributions of each feature to variance in the 1st and 2nd dimension in the PCA shown in Fig. 1B and the Scree Plot shown in Fig. S2A. The 15 features with the greatest contribution to the 1st and 2nd dimension are shown. Dimension 1 is driven by variance in phosphatidylcholines and sphingomyelins, and Dimension 2 by variance in amino acid metabolism.

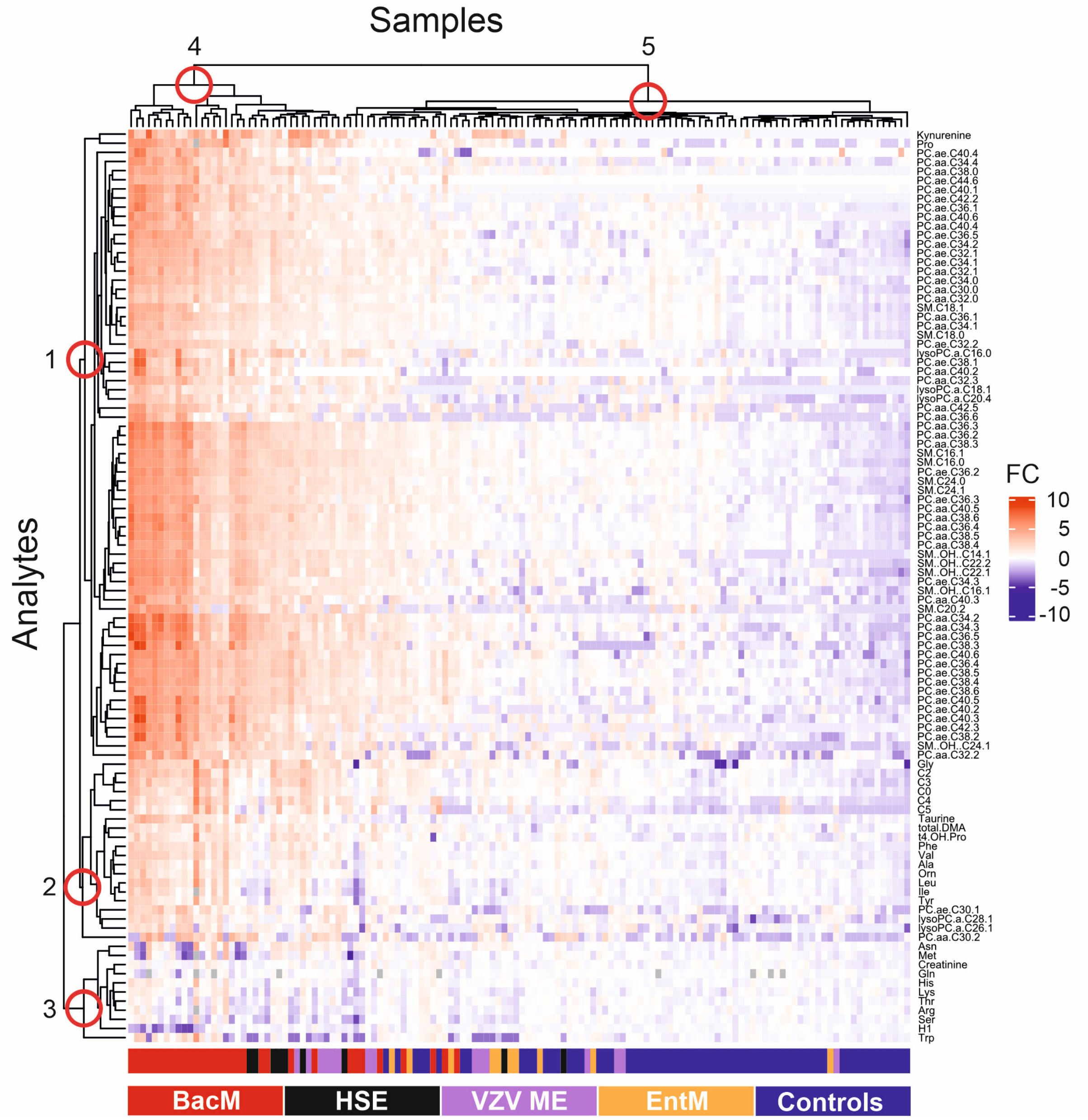


**Figure S3.** Unsupervised hierarchical biclustering analysis using the same metabolite data as for Fig. 1. The samples cluster along the x-axis, and the diagnostic groups are identified by the color code in the legend. Metabolites are clustered along the y-axis. The numbered nodes identify the clades with the following features: 1, predominantly phospholipids; 2 and 3, predominantly amino acids and amino acid metabolites; 4, predominantly bacterial meningitis, also containing some HSV encephalitis and VZV meningitis/encephalitis samples. 5, controls and less inflamed viral samples, e.g. enterovirus meningitis. FC = fold change with respect to mean concentration across all samples.

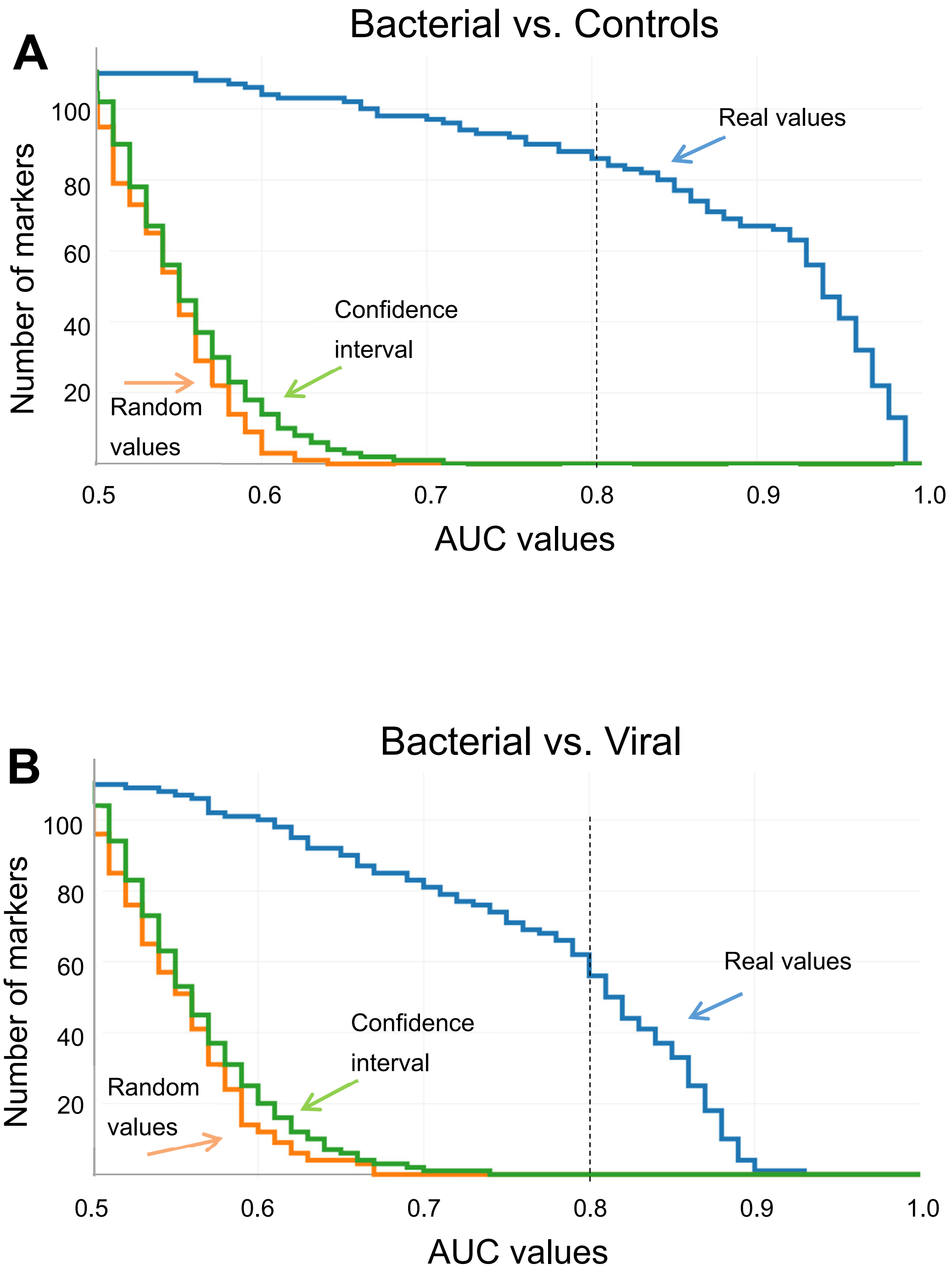


**Figure S4.** HAUCA curve analysis to assess the likelihood of false positive biomarker identification. The number of biomarkers exceeding a given AUC value is plotted on the y-axis. The analysis compares the number of biomarkers identified in the real data set are (blue curve) to those identified in a represented in a random data set (orange curve). The green curve delineates the upper bound 95% CI of the random data set. The vertical line denotes an AUC of 0.8, which is used as cut-off to define biomarkers in the ROC curve analysis shown in Fig. 2. **A.** BacM vs. Controls. **B.** BacM vs. Viral. 95% CI of AUC in the random data set. No markers in the random data set are expected to exceed AUC = 0.8. The likelihood of identifying a marker with AUC ≥ 0.8 in the random data set is essentially zero.

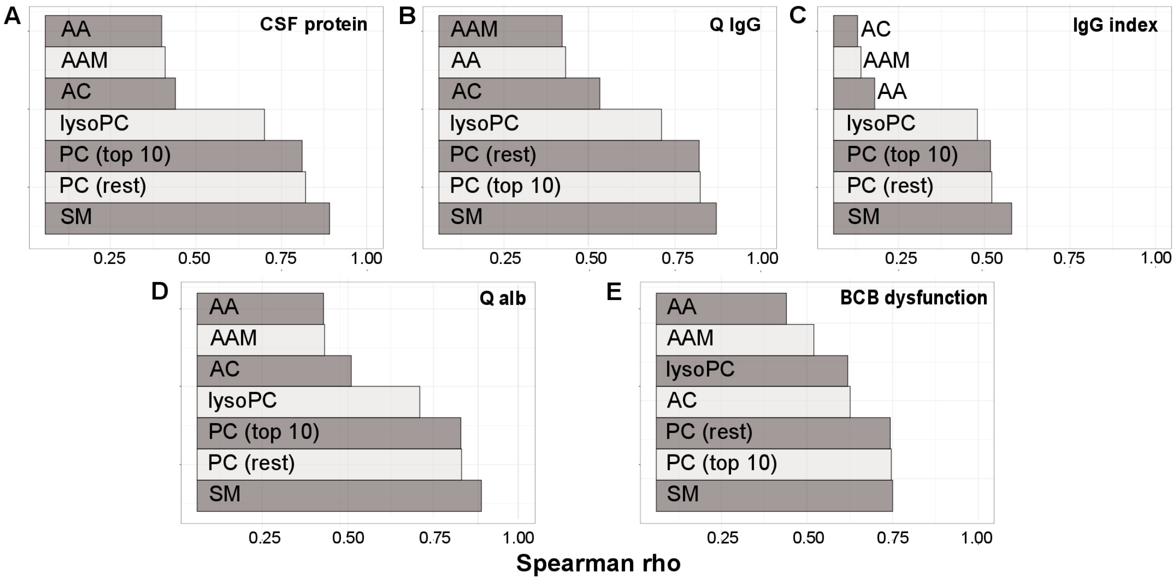


**Figure S5.** Correlations of metabolite subgroups with the CSF and blood parameters not shown in Fig. 3. Values correspond to Pearson correlation coefficient. Abbreviations: AA, amino acids; AAM, amino acid metabolites; AC, acylcarnitines; lysoPC, lysophosphatidylcholines; PC, phosphatidylcholines; SM, sphingomyelins.

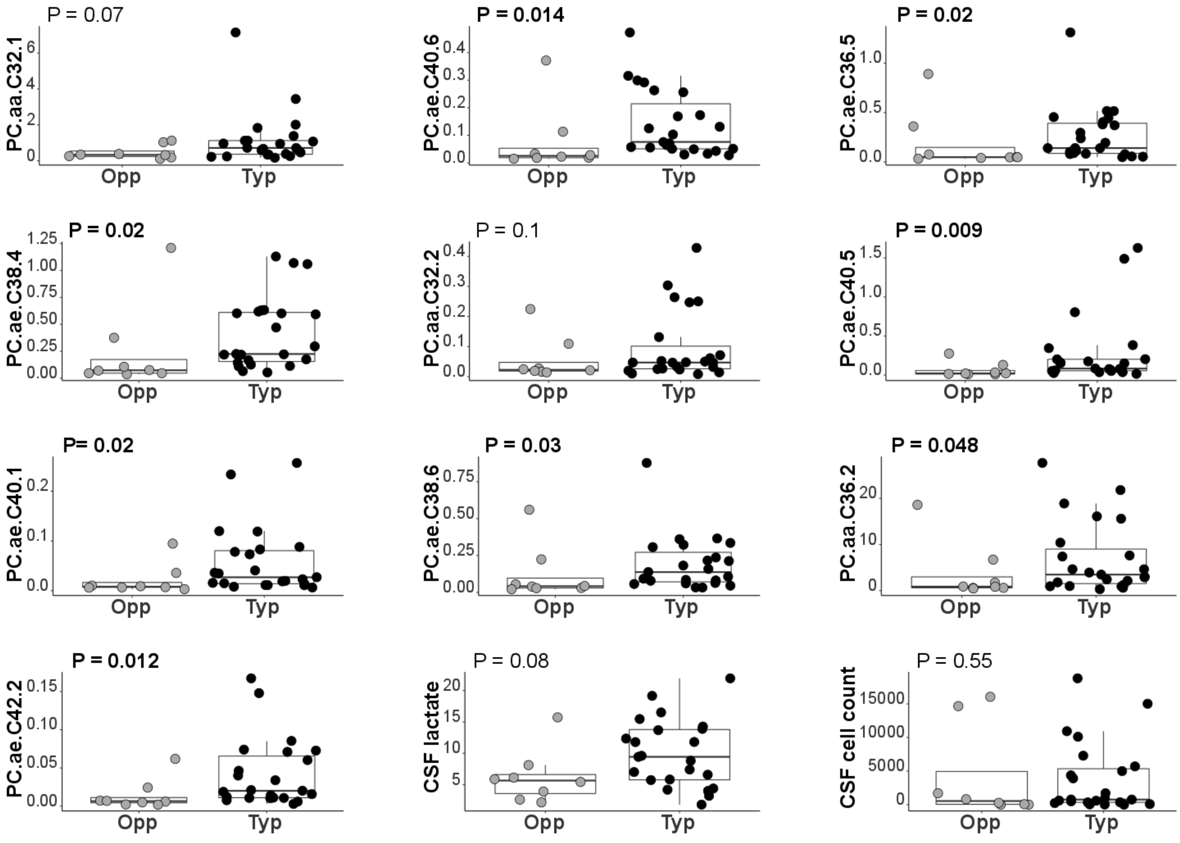


**Figure S6.** Phosphatidylcholine levels are lower in meningitis due to opportunistic vs. typical meningitis bacterial pathogens. Pathogens were classified as “opportunistic” (Opp) or “typical” (Typ) as shown in Table S3. Significance of differences in concentrations of the 10 most accurate phosphatidylcholine biomarkers (Table 2) was assessed by Wilcoxon Rank-Sum test. Differences in CSF lactate and cell count are shown for comparison (lower right). Significant p values (≤ 0.05) are printed in bold.
